# Criterion Validity of Cognitive Reflection for Predicting Job Performance and Training Proficiency: A Meta-Analysis

**DOI:** 10.3389/fpsyg.2021.668592

**Published:** 2021-05-31

**Authors:** Inmaculada Otero, Jesús F. Salgado, Silvia Moscoso

**Affiliations:** Department of Political Science and Sociology, Faculty of Labor Relations, University of Santiago de Compostela, Santiago de Compostela, Spain

**Keywords:** cognitive reflection, job performance, training proficiency, meta-analysis, cognitive intelligence

## Abstract

This article presents a meta-analysis of the validity of cognitive reflection (CR) for predicting job performance and training proficiency. It also examines the incremental validity of CR over cognitive intelligence (CI) for predicting these two occupational criteria. CR proved to be an excellent predictor of job performance and training proficiency, and the magnitude of the true validity was very similar across the two criteria. Results also showed that the type of CR is not a moderator of CR validity. We also found that CR showed incremental variance over CI for the explanation of job performance, although the magnitude of the contribution is small. However, CR shows practically no incremental validity over CI validity in the explanation of training proficiency. Finally, we discuss the implications of these findings for the research and practice of personnel selection.

## Introduction

Cognitive reflection (CR) is one of the most well-known concepts and is based on the view that the human mind operates through two types of cognitive processes known as System 1 (S1) and System 2 (S2). There is high consensus among researchers (Wason and Evans, 1975; Evans and Wason, 1976; Chaiken et al., 1989; Metcalfe and Mischel, 1999; Kahneman and Frederick, 2002, 2005; Stanovich and West, 2002; Strack and Deutsch, 2004; Kahneman, 2011) that S1 is fast, automatic, unconscious and impulsive and operates with little (or no) effort, and that S2 is slow, reflective, and purposeful and requires effort and concentration. Moreover, S2 requires attention, and it is capable of solving complex problems with a high degree of accuracy. On the contrary, S1 tends to use heuristics, biases, and shortcuts to operate quickly and without effort.

Kahneman and Frederick (2002, 2005), Frederick (2005), and Kahneman (2011) defined cognitive reflection as the individual capacity to annul the first impulsive response that the mind offers, and to activate the reflective mechanisms that allow to find a response, make a decision, or carry out a specific behavior. The first impulsive answer is frequently wrong, and cognitive reflection would activate a more reflective and correct answer.

Kahneman and Frederick (2002, 2005), Frederick (2005), and Kahneman (2011) developed the famous cognitive reflection test (CRT) to evaluate individual differences in cognitive reflection. This test consists of three apparently simple arithmetical problems with two alternative answers. Each problem elicits a quick but wrong response. The second answer requires effort, deliberation, and reflection and is the right one. If individuals respond with the first-and-wrong response, they will score low in cognitive reflection. On the contrary, if individuals suppress this first-and-wrong answer in favor of an alternative one, they would be high in cognitive reflection. This test is currently very popular. For example, a search in Google indicates that there are over 38,000 entries with the label “Cognitive Reflection Test,” and Wikipedia also has an entry devoted to the CRT.

Although the CRT (also called 3-item CRT) is the most famous test, other forms of the CRT were developed over the last decades. For instance, some researchers have developed larger CRTs (see, Salgado et al., 2019; Sirota et al., 2020), and others have added new items to the CRT-3 items (e.g., Finucane and Gullion, 2010; Toplak et al., 2014; Primi et al., 2015). Recently, some researchers have shown an interest in the numerical content of the CRT, and they have developed verbal-CRTs (e.g., Thomson and Oppenheimer, 2016; Sirota et al., 2020). These tests also trigger an immediate answer as in the 3-item CRT, and they might involve numbers in their statements, but mathematical calculations are not required in order to find the correct answer.

The relationship between the CRTs and significant cognitive and social criteria has been examined. For instance, empirical research showed that CR predicts achievement in hypothetical decision-making tasks, including intertemporal choice tasks and risky choice tasks. The results suggested that people who score higher in CR showed a preference for delayed-but-bigger gratification against an immediate-but-smaller one (Frederick, 2005; Oechssler et al., 2009). Research also showed that people with higher CR scores tended to display more riskier behaviors in hypothetical financial gain scenarios, even when the expected value of the risky option was lower than the expected value of the safe option (Frederick, 2005). Studies carried out to explore economic behavior employing game theory have suggested that people who scored higher in CR tend to give more accurate answers, deviating less from the normative answers and, consequently, obtaining better results (Moritz et al., 2013; Baghestanian et al., 2015; Corgnet et al., 2015a; Georganas et al., 2015; Noussair et al., 2016; Kocher et al., 2019). It was also found that CR predicted heuristic behavior in decision-making tasks and judgment tasks. For instance, CR predicted the avoidance of several cognitive biases (e.g., base rate neglect, sunk cost effect, conjunction fallacy, and anchoring effect, among others; see for instance, Campitelli and Labollita, 2010; Toplak et al., 2011, 2014) and the resistance to stereotypes and prejudices (Lubian and Untertrifaller, 2013) while making judgments and decisions.

In the organizational domain, the relationships between CR and job performance and training proficiency have also been studied. However, the empirical studies offer a variety of results, some of them showing no relationship between CR and job performance (e.g., Lado et al., 2021) and others showing a moderate correlation (e.g., Corgnet et al., 2015b, 2019; Otero, 2019; Salgado et al., 2019). Research on the relationship between CR and training proficiency also offers an optimistic view, as many studies reported that CR was a relevant predictor of this criterion, although there was also observed variability in the estimates of the relationship (Toplak et al., 2014; Gómez-Veiga et al., 2018; Otero, 2019; Muñoz-Murillo et al., 2020).

When the empirical studies show considerable variability in the relationship between two variables and it is difficult to reach a consensus, meta-analytical techniques offer a potential way of solving the dispute. However, no previous research has meta-analytically examined the relationship between CR and these two critical organizational criteria. Consequently, this article aims to shed further light on the relationships between CR and job performance and training proficiency by providing an estimate of the average correlation between these variables and by examining whether the observed variability is real or artifactual. Moreover, this study also aims to determine whether the type of the CR test moderates the relationship between CR and job performance and training proficiency. Finally, the study also aims to explore whether CR adds validity over cognitive intelligence (i.e., the best predictor of job performance and training proficiency) for predicting both organizational criteria. These four issues are the main goals of this study.

### CR, Job Performance, and Training Proficiency

According to Campbell et al. (1990) and Campbell and Wiernik (2015), job performance refers to any cognitive, psychomotor, motor, or interpersonal behavior, which is controlled by individual, gradable in terms of ability, and relevant for organization goals. Supervisor ratings, work sample test, assessment center ratings, and production records are examples of job performance measures. To our knowledge, few studies have explored the validity of CRT as a predictor of job performance (Morsanyi et al., 2014; Corgnet et al., 2015b; Thoma et al., 2015; Čavojová and Jurkovič, 2017; Otero, 2019; Salgado et al., 2019; Lado et al., 2021). The study carried out by Corgnet et al. (2015b) used a laboratory task as a proxy of job performance. They found that CRT significantly predicted job performance. The study of Čavojová and Jurkovič (2017) used two CRTs, Frederick’s 3-item CRT and a 7-item version. They showed a significant correlation with teacher proficiency. The study of Morsanyi et al. (2014) reported that CR predicted job performance as measured in a laboratory task. Salgado et al. (2019) used an overall rating of an assessment center as the measure of job performance, and Otero (2019) used a work sample test to assess job performance. Both studies found that CR was a predictor of these measures of job performance. Otero (2019) and Salgado et al. (2019) also reported substantial observed validities. More recently, Lado et al. (2021) reported that CR did not significantly correlate with overall job performance ratings. In summary, the literature on CR validity for predicting job performance is relatively scarce, uses two types of CRT (standard version and enlarged versions), and a variety of ways of assessing job performance (e.g., laboratory tasks, assessment center ratings, and work sample tests). The correlations between CR and job performance ranged from 0.05 to 0.47.

Training proficiency refers to the degree of technical skill and competence acquired after a period of education or instruction. Grades, marks, and instructor ratings are examen of training proficiency measures. Previous studies have shown that CR is a relevant predictor of several measures of training proficiency. For example, Muñoz-Murillo et al. (2020) found that the CRT substantially correlated with grade point average (GPA), and Toplak et al. (2014) also reported a significant validity for predicting self-reported GPA. Thomson and Oppenheimer (2016) found that a verbal CRT and a numerical CRT were valid predictors of GPA. Other studies conducted by Morsanyi et al. (2014); Otero (2019), and Salgado et al. (2019) also found substantial correlations. However, Corgnet et al. (2015b) did not found correlation between CRT and GPA.

Other indicators of training proficiency have also been explored. For example, research has shown that CRT was a predictor of mathematical achievement (Gómez-Chacón et al., 2014; Gómez-Veiga et al., 2018), physics qualifications (Lindeman and Svedholm-Häkkinen, 2016), scores in the math and language section of the German A-level exam (Lohse, 2016), statistics exam qualifications (Primi et al., 2017), and educational level (Yılmaz and Sarıbay, 2016, 2017; Skagerlund et al., 2018). However, CRT was not a predictor of school attendance (Primi et al., 2017).

Despite the fact that the number of studies that have examined the relationship between CR and job performance and training proficiency is relatively low, the majority of the studies found that the CR test predicts both organizational criteria. However, there is substantial variability in the magnitude of the correlations. There are both methodological and theoretical reasons that might explain the variability. Artifactual errors (i.e., sampling error, measurement error, and range restriction) might explain a part of or the totality of the observed variability. Another source of variation in the correlations might be that the studies used different CRTs, with different reliabilities. It was repeatedly found that the CRT-3 showed low internal consistency and that the enlarged version of the CRT and the new CRTs have higher reliability than the original one (see Otero, 2019; Salgado et al., 2019; Otero et al., 2021 for more details). Reliability (measurement error) produces two artifactual effects. First, it attenuates the magnitude of the correlations. Second, it introduces error variance into the distributions of correlations. The third source of variation might be that the studies used a variety of estimates of job performance and training proficiency, which have different psychometric properties (e.g., construct validity and reliability). Based on the research discussed above, we posit the following research questions:

*Research Question 1:* What is the correlation between CR and job performance?

*Research Question 2:* What is the correlation between CR and training proficiency?

*Research Question 3:* Is the observed variability in the correlation between CR and job performance and training proficiency real or artifactual?

*Research Question 4:* Does the type of CRT moderate the relationship between CR and job performance and training proficiency?

### Incremental Validity of CR

Some studies have examined the incremental validity of CR over other variables for predicting training proficiency and job performance, although the number of studies is small. Cognitive intelligence (CI) occupies a special place in these sorts of studies because many meta-analyses have indicated that CI is the best predictor of job performance and training proficiency and that CI generalizes the validity across organizations, jobs, and samples (e.g., Hunter and Hunter, 1984; Kuncel et al., 2001, 2005; Salgado et al., 2003; Bertua et al., 2005; Richardson et al., 2012; Salgado and Moscoso, 2019). For instance, a recent meta-analysis conducted by Salgado and Moscoso (2019) found that CI showed an average true validity of 0.44 and 0.62 for predicting job performance and training proficiency, respectively.

Two studies (Otero, 2019; Salgado et al., 2019) tested if CR showed incremental validity of CR over CI for predicting job performance and training proficiency. Salgado et al. (2019) found that the joint validity of CR and CI for predicting job performance was higher than the validity of CI alone, but the incremental validity was small (Δ*R*^2^ = 0.03). Likewise, Otero (2019) found that CR added validity over CI, but again the incremental validity was small (Δ*R*^2^ = 0.01).

The incremental validity findings concerning the training proficiency are less robust than the findings for job performance. Salgado et al. (2019) showed that CR added validity over CI in the prediction of training proficiency, although the incremental validity was also small (Δ*R*^2^ = 0.01). Meanwhile, Otero (2019) found that CR only added validity over CI in the prediction of GPA (Δ*R*^2^ = 0.01), but not other academic outcomes such as high school grades and college admission scores. Toplak et al. (2014) reported that CR did not add validity over CI in the prediction of GPA. Hence, the fifth research question is:

*Research Question 5:* Does CR add validity over CI for predicting job performance and training proficiency?

## Method

### Literature Search

We used several strategies to review the literature. The search covered the studies carried out from September 2005 to December 2019. First, we conducted electronic searches in the ERIC database and in the Google Scholar meta-database using the keywords “Cognitive Reflection” and “Cognitive Reflection Test.” Second, we carried out an article-by-article search in the *Journals of Applied Cognitive Psychology, Cognition, Cognitive Science, Frontiers in Psychology; Journal of Applied Research; Journal of Behavioral Decision Making; Journal of Economic Behavior and Operation; Journal of Experimental Psychology: General; Journal of Operations Management, Judgment and Decision Making, Memory and Cognition, Mind and Society, Production and Operations Management; The Journal of Economic Perspectives;* and *The Journal of Socio-Economics; Journal of Behavioral and Experimental Economics, and Thinking and Reasoning*. Third, we checked the reference lists of the papers to identify articles not covered in the abovementioned strategies. Fourth, we contacted researchers of the CR articles to obtain new studies or supplementary information.

**FIGURE 1:**
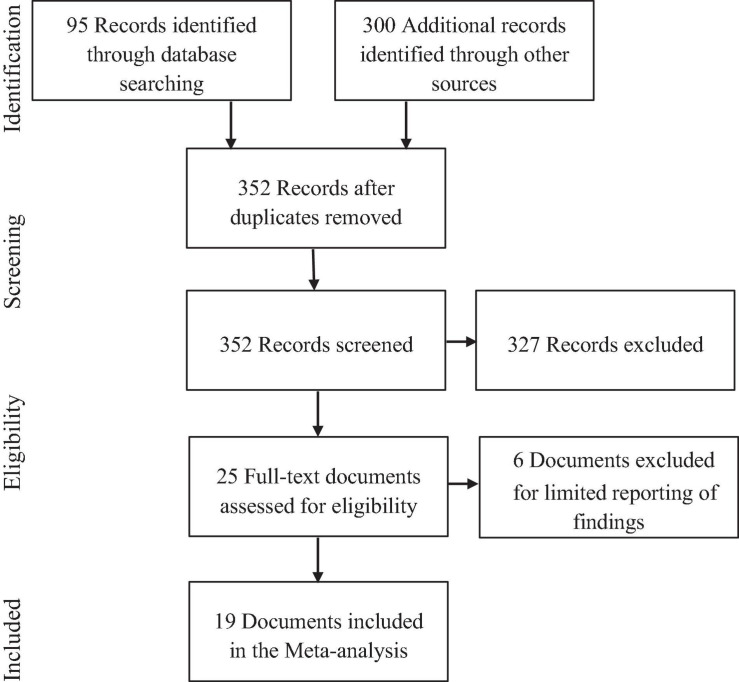
PRISMA flowchart of information through the different phases of a systematic review.

A total of 95 records were identified through the database searches, and 300 additional records were identified through the other three strategies. The content of these 395 records was examined, and the studies that did not provide correlations or data to calculate the correlations between CR and job performance and training proficiency were discarded. The final database consisted of 19 documents. A PRISMA flowchart of information through the different phases of this systematic review appears in Figure 1.

### Inclusion Criteria and Decision Rules

The content of each record was examined to determine their inclusion in the meta-analysis. We included the studies that provided a correlation or other data that allowed us to estimate the relationship between CR and job performance and training proficiency.

Regarding job performance, we included the correlations between CR and several measures of this occupational criterion. For instance, we included experimental tasks as a proxy of job performance (see Morsanyi et al., 2014; Corgnet et al., 2015b) and the overall performance ratings (see Otero, 2019; Salgado et al., 2019).

For training proficiency, we included the measures of GPA and exam grades. In both cases, the official and the self-reported scores were included. Moreover, knowledge tests required for admission to specific courses were also included as training proficiency measures.

We also included studies where CR was measured with the 3-item CRT or with other CRTs. As other CRTs, we included the tests composed entirely of new items and those which include some of Frederick’s original items, and the CRTs composed entirely of verbal items or combined with numerical ones. The CRTs composed of three parallel items of the 3-item CRT were not considered as other CRTs, given that the difference between those resides in the nouns that involved (e.g., the bat and ball are substituted for TV and CD; see, for instance, Mata et al., 2013).

When the studies reported, from the same sample, effect sizes administering the 3-item CRT and other CRTs, we included the effect size obtained from 3-item CRT. Then, we also examined the relationship between CR and both organizational criteria according to the CRT type (i.e., 3-item CRT or other CRTs). A meta-analysis using only verbal CRTs could not be conducted because we found hardly any studies.

Based on the above, the meta-analysis on the relationship between CR and job performance was conducted with an accumulated sample size of 1,508 subjects and 7 effects sizes, and the meta-analysis on the relationship between CR and training proficiency was carried out with an accumulated sample of 3,705 subjects and 15 effects sizes. In accordance with MARS and PRISMA guidelines, the primary studies included in the meta-analyses and the relevant information about them (i.e., sample size, observed correlation, predictor reliability, criterion reliability, and the type of the CR test) can be found in Tables 1, 2 for job performance and training proficiency, respectively.

**TABLE 1:** Studies included in the meta-analysis of CR and job performance.

**References**	**Type of CRT**	***N***	***r*_*xy*_**	***r*_*xx*_**	***r*_*yy*_**
^*a*^Čavojová and Jurkovič, 2017	3-items	164	0.24	0.65	0.80
	7-items	164	0.34	0.76	0.80
Corgnet et al., 2015b	3-items	264	0.34	–	–
Corgnet et al., 2019	3-items	224	0.31	–	–
^*a*^Lado et al., 2021	3-items	245	0.01	0.71	0.90
	13-items	245	0.05	0.79	0.90
^*a*^Otero, 2019	3-items	475	0.30	0.58	0.96
	13-items	475	0.34	0.76	0.96
Salgado et al., 2019	13-items	100	0.33	0.82	0.84
Thoma et al., 2015	7-items	36	0.15	–	–

**N*, sample size; *r*_*xy*_, observed correlation; *r*_*xx*_, internal consistency reliability of cognitive reflection measure; *r*_*yy*_, internal consistency reliability of job performance.*

*^*a*^The observed correlations are provided from the same sample but using different CR test.*

**TABLE 2:** Studies included in the meta-analysis of CR and training proficiency.

**References**	**Type of CRT**	***N***	***r*_*xy*_**	***r*_*xx*_**	**Study**
Corgnet et al., 2015b	3-items	264	0.04	–	
Gómez-Chacón et al., 2014	5-items	56	0.25	–	
Gómez-Veiga et al., 2018	3-items	51	0.32	–	
Insler et al., 2015	3-items	364	0.31	–	
Lindeman and Svedholm-Häkkinen, 2016	3-items	258	0.34	–	
Lohse, 2016	3-items	284	0.27	–	
Morsanyi et al., 2014	3-items	328	0.45	0.57	Study 1
	3-items	184	0.29	0.68	Study 2
Muñoz-Murillo et al., 2020	3-items	189	0.25	–	
^*a*^Otero, 2019	3-items	898	0.16	0.58	Study 3
	13-items	898	0.15	0.78	Study 3
^*a*^Primi et al., 2017	3-items	124	0.17	–	
	6-items	124	0.22	0.76	
Stupple et al., 2017	3-items	133	0.26	–	
^*a*^Tamas, 2019	3-items	269	0.61	–	
	10-items	269	0.52	–	
^*a*^Thomson and Oppenheimer, 2016	3-items	143	0.33	0.62	
	7-items	143	0.37	0.71	
^*a*^Toplak et al., 2014	3-items	160	0.23	–	
	7-items	160	0.25	0.72	

**N*, sample size; *r*_*xy*_, observed correlation; *r*_*xx*_, internal consistence reliability of cognitive reflection measure.*

*^*a*^The observed correlations are provided from the same sample but using different CR test.*

### Meta-Analytic Method

The Schmidt and Hunter’s (2015) psychometric meta-analytic method of correlations was used to analyze the data. This method estimates the true score correlation and the operational correlation between predictor and criterion variables, correcting the mean observed correlations for the artifactual effects. It also estimates the proportion of observed variance (in findings across studies) that is due to the artifacts. The artifacts controlled for in the current meta-analysis were sampling error, measurement error in the predictor variable (i.e., CR), and measurement error in the criterion variable (i.e., job performance and training proficiency). However, because the primary studies rarely provided all the information required to individually correct the observed correlation, it was necessary to create empirical distributions for each artifact.

We developed reliability distributions for predictor (i.e., CR) and criterion (i.e., job performance and training proficiency) variables. The distributions were based upon internal consistency coefficients reported in the primary studies (Salgado et al., 2016). In the case of training proficiency, we used the reliability distribution developed by Cuadrado et al. (2021). This distribution was created by 7 coefficients of internal consistency (from 0.78 to 0.98), and the obtained average reliability coefficient was 0.87 (*SD* = 0.06). The mean and the standard deviation of the reliability distributions appear in Table 3.

**TABLE 3:** Predictor and criteria reliability distributions.

	***K***	***r*_xx_**	***SD***	**Min.–Max.**
**Predictor reliability**				
CR–job performance	4	0.69	0.10	0.58–0.82
CR–training proficiency	5	0.64	0.08	0.58–0.76
**Criteria reliability**				
Job performance	4	0.88	0.07	0.80–0.96
Training proficiency^*a*^	7	0.87	0.06	0.78–0.98

**K*, number of cases; *r*_*xx*_, average internal consistency reliability; *SD*, the standard deviation of *r*_*xx*_; Min.–Max., minimum and maximum value of *r*_*xx*_; CR, cognitive reflection.*

*^*a*^Reliability distribution developed by Cuadrado et al. (2021).*

Finally, we corrected the observed mean correlation for measurement error in the criterion variable to obtain the operational correlation (which is relevant for personnel and academic decisions), and we corrected the operational correlation for measurement error in the predictor variable to obtain the true score correlation (which is relevant for testing theoretical relationships). The analyses were carried out by Schmidt and Le’s statistical software (Schmidt and Le, 2014).

## Results

### Meta-Analyses of the CR Validity for Predicting Job Performance and Training Proficiency

Table 4 presents the results of the meta-analysis of the CR validity for predicting job performance. The table, from left to right, presents the number of validity coefficients (*K*), the total sample size (*N*), the observed average correlation (*r*), the observed variance of *r* (S^2^*_*r*_*), the standard deviation of *r* (*SD*_*r*_), the operational validity (*r*_*op*_), the true validity (ρ), the standard deviation of ρ (*SD*_ρ_), the 90% credibility value (90% CV), the 95% confidence interval of ρ (95% CI), and the percentage of variance explained by artifacts (%VE).

**TABLE 4:** Meta-analytic results of the CR validity for predicting job performance.

	**Bare-bones meta-analysis**					**Psychometric meta-analysis**					
	***K***	***N***	***r***	***S^2^_*r*_***	***SD*_*r*_**	***r*_*op*_**	**ρ**	***SD*_ρ_**	***90% CV***	***95% CI***	***%VE***
CR–job performance	7	1,508	0.28	0.0119	0.106	0.31	0.36	0.106	0.22	0.26–0.46	38
CR 3-item	5	1,372	0.25	0.0129	0.114	0.27	0.33	0.129	0.17	0.20–0.47	26
Other CR tests	5	1,020	0.26	0.0154	0.124	0.29	0.32	0.128	0.16	0.19–0.46	28

**K*, the number of validity coefficients; *N*, the total sample size; *r*, the observed average validity weighted by the study sample size; $S_{r}^{2}$, the observed variance of *r*; *SD_r*, the standard deviation of *r*; *r*_op_, the operational validity; ρ, the true validity; *SD*_ρ_, the standard deviation of ρ; 90% CV, the 90% credibility value; 95% CI, the 95% confidence interval of ρ; %VE, the percentage of observed variance explained by artifacts.*

The meta-analytical results show that the observed validity, the operational validity, and the true correlation of CR are 0.28, 0.31, and 0.36, respectively. The values indicate that the CR is a valid predictor of job performance and that the magnitude of the validity is of medium size. Sampling error, CR reliability, and job performance reliability explain 38% of the variance, which suggests that a search for a moderator is in order. The 90% credibility value is notably different from zero, which provides evidence of validity generalization.

Therefore, this first meta-analysis permits to conclude that CR predicts job performance efficiently and that validity generalizes across samples, CR measures, and job performance measures. However, the amount of explained variance is small (13%).

A potential moderator of the CR validity is the type of CRT. In the data set, we observed that studies could be classified into two main groups, a first group of studies that uses the 3-item CRT and a second group of studies that uses other CRTs. Based on this classificatory scheme, we conducted new meta-analyses for the combinations of the type of CRTs and job performance.

Regarding the effects of the type of CRT on validity magnitude and variability, we found that although the observed validity and the operational validity of the 3-item CRT are slightly lower than the respective values for the studies that used CRTs with a higher number of items (0.25 and 0.27 vs. 0.26 and 0.29), the true correlation is slightly higher for 3-item CRT studies than for the studies that used CRT measures with more items (0.33 vs. 0.32). These differences have no practical relevance, and they indicate that the measurement error in the 3-item CRT is higher than that in the other CRTs. Also, the standard deviations and the percentage of explained variance are practically the same for the two groups of studies. Consequently, these results suggest that the type of CRT does not moderate the CR validity for predicting job performance.

Table 5 shows the results of CR validity for predicting the training proficiency. The estimates for the observed validity, operational validity, and true correlation are 0.27, 0.29, and 0.37, respectively. The 90% CV is 0.16, which indicates that CR generalizes the validity across samples, CR measures, and training proficiency measures. Therefore, as happened with job performance, this analysis allows to conclude that CR is also a robust predictor of the training proficiency.

**TABLE 5:** Meta-analytic results of the CR validity for predicting training proficiency.

	**Bare-bones meta-analysis**					**Psychometric meta-analysis**					
	***K***	***N***	***r***	***S^2^_*r*_***	***SD*_*r*_**	***r*_*op*_**	**ρ**	***SD*_ρ_**	***90% CV***	***95% CI*_ρ_**	***%VE***
CR–training proficiency	15	3,705	0.27	0.0190	0.138	0.29	0.37	0.168	0.16	0.28–0.47	19
CR 3-item	14	3,649	0.27	0.0193	0.139	0.29	0.37	0.170	0.16	0.28–0.47	18
Other CR tests	6	1,650	0.25	0.0185	0.136	0.26	0.31	0.152	0.11	0.17–0.44	18

**K*, the number of validity coefficients; *N*, the total sample size; *r*, the observed average validity weighted by the study sample size; $S_{r}^{2}$, the observed variance of *r*; *SD_r*, the standard deviation of *r*; *r*_op_, the operational validity; ρ, the true validity; *SD*_ρ_, the standard deviation of ρ; 90% CV, the 90% credibility value; 95% CI, the 95% confidence interval of ρ; %VE, the percentage of observed variance explained by artifacts.*

Concerning the observed variability, the three artifactual errors considered in this meta-analysis explain 19% of the observed variance, which suggests that potential moderators can explain the remaining variance.

As in the case of the job performance criterion, we examined whether the type of CRT moderates the validity of CR for predicting the training proficiency. To this regard, we found that although the estimate for the observed validity, operational validity, and true correlation of the 3-item CRT is slightly higher than the respective values for the studies that used CRTs with a higher number of items (0.27, 0.29, and 0.37 vs. 0.25, 0.26, and 0.31, respectively), these differences have no practical relevance, and they indicate that both shorter and longer CR tests are similarly efficient for predicting training proficiency. Also, the standard deviations and the percentage of variance explained by artifacts are very similar for the two groups of studies. Consequently, these results suggest that the type of CRT does not moderate the CR validity for predicting the training proficiency.

### Incremental Validity of CR Over CI for Predicting Job Performance and Training Proficiency

Research Question 5 asked whether the CR shows incremental validity over CI for predicting the two criteria examined in this meta-analysis. Consequently, we carried out hierarchical multiple regression analyses. The first step consisted of estimating the validity, beta, and explained variance of CI for predicting both criteria. In the second step, we entered CR to estimate the multiple *R*, *R*^2^, respective betas for CI and CR, and the incremental validity of CR over CI. To conduct these analyses, we used a matrix of true correlations following Schmidt and Hunter’s (2015) advice.

In this regard, we created two matrices with the correlations between CI, CR, and the two criteria. We used the values found in this meta-analysis as the correlations between CR and job performance (ρ = 0.36) and CR and training proficiency (ρ = 0.37). We used the correlation found by the meta-analysis of Otero et al. (2021) as an estimate of the relationship between CI and CR (ρ = 0.53). Finally, we used the validities reported by Salgado and Moscoso (2019, **Table 12**) for the true correlations between CI and job performance (ρ = 0.44) and CI and training proficiency (ρ = 0.62). The harmonic average of all sample sizes was estimated to determine the sample size entered in the regression analysis. According to Judge et al. (2007), the harmonic average is the best estimator of the regression analysis sample size when the purpose is to generalize the results to the population. We decided to follow this recommendation, and the harmonic sample size for the analysis was 4,427.

Table 6 reports the results for the prediction of job performance. As can be seen, CR shows a small (2.5%) but statistically significant incremental validity over CI. The validity changes from 0.440 to 0.465, and the beta weights for CI and CR are also statistically significant. Interestingly, when the two variables are entered in the regression equation, the beta for CI diminishes from 0.440 to 0.347 (a decline of 21%), which indicates that CR is a relevant predictor of job performance, although its contribution is small.

**TABLE 6:** Incremental validity of the CR over the CI for predicting job performance.

	**β**	***R***	***R*^2^**	***p***	**Δ*R***
**Step 1**					
CI	0.440	0.440	0.194	0.000	
**Step 2**					
CI	0.347	0.465	0.216	0.000	0.025
CR	0.176				

**N* = 4,427. All β values are significant at *p* < 0.001 value. CI, cognitive intelligence; CR, cognitive reflection; β, standardized regression weights; *R*, multiple regression; *R*^2^, squared multiple correlation; Δ*R*, incremental validity of the CR over and above the CI.*

Table 7 reports the results for the prediction of training proficiency when CR supplements CI. As can be seen, the incremental validity of CR is minimal (0.002) and practically irrelevant. The validity changes from 0.620 to 0.622, although the beta for CR is statistically significant. When the two variables are entered in the regression equation, the beta for CI diminishes from 0.620 to 0.589 (a decline of 5%), which indicates that CR is not a relevant predictor of training proficiency.

**TABLE 7:** Incremental validity of the CR over the CI for predicting training proficiency.

	**β**	***R***	***R*^2^**	***p***	**Δ*R***
**Step 1**					
CI	0.620	0.620	0.384	0.000	
**Step 2**					
CI	0.589	0.622	0.387	0.000	0.002
CR	0.058				

**N* = 4,427. All β values are significant at *p* < 0.001 value. CI, cognitive intelligence; CR, cognitive reflection; β, standardized regression weights; *R*, multiple regression; *R*^2^, squared multiple correlation; Δ*R*, incremental validity of the CR over and above the CI.*

As a whole, these incremental validity analyses show that CR is a useful predictor of job performance but not relevant for predicting the training proficiency.

## Discussion

This meta-analytic study examined the current empirical evidence on the relationship between CR and job performance and training proficiency. Moreover, the study used meta-analytically developed correlation matrices to determine the incremental validity of CR over the CI for predicting these two criteria. The meta-analysis aimed to answer five research questions: (1) What is the correlation between CR and job performance? (2) What is the correlation between CR and training proficiency? (3) Is the observed variability in the correlation between CR and job performance and training proficiency real or artifactual? (4) Does the type of CR test moderate the relationship between CR and job performance and training proficiency? (5) Does CR add validity over CI for predicting job performance and training proficiency? In examining the findings of the primary studies with meta-analytical methods, the current research made three unique contributions to the understanding of the relationships between CR, job performance, and training proficiency and the degree of CR validity generalization and incremental validity.

The first unique contribution has been to show that the CR predicts both job performance and training proficiency. The magnitude of the true correlations is similar or even higher than other widely used predictors of these two criteria, such as personality dimensions, emotional intelligence, specific cognitive abilities, work sample tests, assessment centers, situational and judgment tests, biodata, and social network websites (for instance, Barrick and Mount, 1991; Ones et al., 1993, 2012; Salgado, 1997, 2017; Van Rooy and Viswesvaran, 2004; Joseph and Newman, 2010; Judge et al., 2013; Salgado and Tauriz, 2014; Whetzel et al., 2014; Alonso et al., 2015; Salgado and Lado, 2018; Aguado et al., 2019; Herde et al., 2019; Morillo et al., 2019; Ryan and Derous, 2019; Salgado and Moscoso, 2019; García-Izquierdo et al., 2020; Golubovich et al., 2020; Otero et al., 2020).

The second contribution has been to show that the type of CRTs (i.e., shorter and longer) does not significantly moderate the magnitude of the true correlations. Despite the fact that the short CRT has lower reliability and other psychometric weaknesses, the validity, when corrected for measurement error in CR and criterion, is very similar for this test and other CRTs created to overcome these psychometric limitations.

The third unique contribution has been to show that CR contributes over CI to the explanation of the variance of job performance, although the magnitude of the contribution is small. Despite this fact, the incremental validity and the significant beta indicate that CR should be considered independently from CI when the interest is the prediction of job performance. In the case of training proficiency, CR shows practically no incremental validity over CI validity. The results of multiple regression analysis have indicated that CI supplemented by CR explains the same amount of training proficiency variance as CI alone. This finding contrasts with previous results, which suggested that CR could add validity over CI for predicting GPA (e.g., Otero, 2019; Salgado et al., 2019).

The findings of this meta-analysis have theoretical and practical implications. From the theoretical point of view, the results suggest that CR and CI can be conceptualized as related but different cognitive constructs because they contribute independently to the explanation of job performance.

From a practical point of view, the findings also have relevant implications. On the contrary, as CR correlates significantly with job performance, CRTs could be included among the procedures used for personnel selection, particularly when CI tests were not included in the batteries of selection procedures. CRTs are not a substitute for CI tests, because they represent different constructs, but the criterion validity of the CRTs was of sufficient magnitude for them to be used as an alternative test in the cases in which CI measures are not possible. On the contrary, as CRTs correlate with training proficiency, they can also be used for making decisions in an academic context, for instance, CRT could be used in admission processes (e.g., university studies, training courses, and scholarships).

Like all studies, the present one also has some limitations that must be mentioned. The first limitation is that the number of primary studies is too small for conducting more fine-grain moderator analyses. For example, we were not able to examine whether job complexity, a relevant moderator of CI validity, is also a moderator of CR validity. The scarcity of studies is also relevant in connection with the measurement of job performance and training proficiency. For example, supervisory performance ratings and instructor ratings, probably the most frequently used measures of these two criteria, were missing in the current database. Therefore, we suggest that future studies include these two types of measures.

## Conclusion

This research provided the first meta-analysis, which examined the relationships between CR and job performance and training success, showing that CR is an excellent predictor of these two organizational criteria. Moreover, this research showed that CR and CI are not empirically redundant because CR adds validity over CI validity for predicting job performance, although not to predict training proficiency. The results also showed that the type of CRT does not significantly moderate the magnitude of the relationship between CR and job performance and training proficiency. Future research should be conducted to expand the contributions of this study and to clarify some issues, which were not examined here.

## Data Availability Statement

The original contributions presented in the study are included in the article/supplementary material, further inquiries can be directed to the corresponding author.

## Author Contributions

All authors listed have made a substantial, direct and intellectual contribution to the work, and approved it for publication.

## Conflict of Interest

The authors declare that the research was conducted in the absence of any commercial or financial relationships that could be construed as a potential conflict of interest.
